# Better muscle strength can decrease the risk of arthralgia and back &joint stiffness in Kurdish men; a cross-sectional study using data from RaNCD cohort study

**DOI:** 10.1186/s12891-020-03712-5

**Published:** 2020-10-16

**Authors:** Yahya Pasdar, Behrooz Hamzeh, Shima Moradi, Sahar Cheshmeh, Farid Najafi, Mehdi Moradinazar, Mohammad Bagher Shamsi, Ebrahim Shakiba

**Affiliations:** 1 Department of Nutritional Sciences, Research Center for Environmental Determinants of Health (RCEDH), Health Institute, Kermanshah University of Medical Sciences, Kermanshah, Iran; 2 School of Public Health, Research Center for Environmental Determinates of Health (RCEDH), Kermanshah University of Medical Sciences, Kermanshah, Iran; 3 Department of Nutritional Sciences, Research Center for Environmental Determinants of Health (RCEDH), Health Institute, Kermanshah University of Medical Sciences, Kermanshah, Iran; 4 Student of Research Committee, School of Nutritional Sciences and Food Technology, Kermanshah University of Medical Sciences, Kermanshah, Iran; 5 School of Public Health, Communing Developmental and Health Promotion Research center, Kermanshah University of Medical Sciences, Kermanshah, Iran; 6 Behavioral Disease Research Center, Kermanshah University of Medical Sciences, Kermanshah, Iran; 7 Rehabilitation and Sports Medicine Department, Kermanshah University of Medical Sciences, Kermanshah, Iran; 8 Social Development and Health Promotion Research Center, Kermanshah University of Medical Sciences, Kermanshah, Iran

**Keywords:** Muscle strength, Low back pain, Arthralgia, Back stiffness, Joint stiffness

## Abstract

**Background:**

Musculoskeletal disorders can reduce the quality of life and work capacity. The study assessed handgrip strength (HGS) in relation to low back pain and arthralgia in Kurdish men.

**Methods:**

This cross-sectional study was conducted using data from Ravansar non-communicable diseases (RaNCD) cohort study on 2164 men aged 35–65 years. HGS was measured using a hand-held hydraulic handgrip dynamometer. Low back pain, arthralgia, and joint stiffness were evaluated by the RaNCD cohort study physician using a standard questionnaire.

**Results:**

The results showed that 21.39 and 24.58% of studied participants had low back pain and arthralgia, respectively. Among the participants with low back pain, 14.5% had back stiffness, and among those with arthralgia, 12.8% had joint stiffness. The mean of HGS in participants with arthralgia and back & joint stiffness was significantly less than those without these disorders (*P* < 0.001, *P* = 0.05, and *P* = 0.005, respectively). Multiple-adjusted OR and 95% confidence intervals (CI) for arthralgia and back and joint stiffness across muscle strength showed the HGS increase to be associated with a lower risk of arthralgia and back &joint stiffness, but not low back pain.

**Conclusions:**

Higher HGS was associated with a lower risk of arthralgia and back & joint stiffness. However, there was no association between HGS and low back pain. Exercise and adherence to proper nutrition are suggested to enhance muscle strength in order to reduce musculoskeletal pain.

## Introduction

One of the most common musculoskeletal problems is low back pain, which affects people’s quality of life [1]. Non-specific low back pain is the main cause of back pain; however; low back pain is usually caused by disc herniation, lumbar stenosis, trauma, muscle strain, lumbar spondylosis, spine and kidney infections, certain cancers, endometriosis, arthritis, and ankylosing spondylitis [2, 3]. Results from a systematic review and meta-analysis on 41 studies have shown that the incidence of low back pain is about 25% among people who have experienced for the first time, without considering community or occupation [4]. In another systematic review and meta-analysis by Fatoye in 2019 on routinely collected data, the incidence range of low back pain has been reported 0.024–7.0% [5]. It should be noted that since many musculoskeletal disorders are not reported, the number of people with these disorders is much higher in the community [6]. Many studies have reported that the prevalence of musculoskeletal disorders is higher in older men and women than young [6, 7]. However, the peak prevalence of symptoms has been reported between the ages of 40 and 69 [4]. Generally, low back pain is the major reason for immobility, as well as decreased physical function and work capacity that is important, especially in men as the main workforce [2].

Arthralgia, also known as joint pain, is the pain in the area of joints, usually being a subjective symptom of arthritis [8]. Inflammation plays a major role in causing chronic joint pain [9]. In this disorder, joint pain and stiffness occur mainly in the joints of the fingers, wrists, legs, and, in more severe cases, elbows, shoulders, knees, neck, and hip [10].

Handgrip strength (HGS) is a convenient and practical method in clinical and epidemiological studies to reflect musculoskeletal function, and its physical and nutritional status [11, 12]. This reliable method can reflect overall muscle strength and nutritional status [13]. Optimal muscle strength is associated with a lower risk of non-communicable diseases (NCDs), such as cardiovascular and diabetes, and overall mortality [11, 14]. According to the high prevalence of musculoskeletal pain, we hypothesize that the recognition of the association between muscle strength and the risk of low back pain and arthralgia could provide suggestions for modifying and improving lifestyle to reduce these pains. Therefore, the present study aims to evaluate the relationship between HGS and the risk of low back pain and arthralgia in Kurdish men in Ravansar non- communicable diseases (RaNCD) cohort study.

## Material and methods

### Study design and setting

The present cross-sectional study was conducted on baseline data of men enrolled in RaNCD cohort study. The RaNCD cohort study is the first Kurdish population-based study on 10,059 Kurdish participants (4770 men and 5289 women) aged 35–65 years living in Ravansar city, Kermanshah province, Western- Iran since 2014. It is one of 18 studies developed by the PERSIAN (Prospective Epidemiological Research Studies in Iran) mega cohort study that was approved by the Ethics Committees in the Ministry of Health and Medical Education, the Digestive Diseases Research Institute, Tehran University of Medical Sciences, Iran. The details of this study were described in previous studies [15, 16]. This cohort study was approved by the Ethics Committee of Kermanshah University of Medical Sciences (No: KUMS.REC.1394.318).

### Participants

**Fig. 1:**
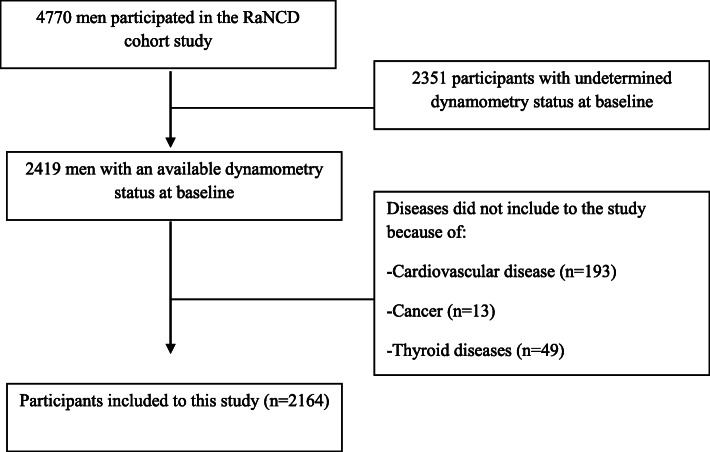
Flowchart of the samples selection

Inclusion criteria in this study were men that their muscle strength were measured in the first phase of the RaNCD cohort study. Since muscle mass decreases in cardiovascular diseases, cancer, and thyroid diseases [17–20], we did not include people with these diseases (Fig. 1).

### Data sources/measurements

#### Anthropometric indices

In the study site in Ravansar, InBody 770 device (Inbody Co, Seoul, Korea) was applied to measure the weight of participants with the least clothing and without shoes. In order to measure height, the automatic stadiometer BSM 370 (Biospace Co., Seoul, Korea) was used in a standing position without shoes with a precision of 0.1 cm. Body mass index (BMI) was calculated using this formula: weight (kg)/ (height)^2^ (meter)^2^. Waist circumference was measured by non-stretched and flexible tape in standing position at the level of the iliac crest three times, and the average was reported.

#### Hand grip strength

A hand-held hydraulic handgrip dynamometer (Model SH5003; SAEHAN Corporation, Masan, Korea) was applied to measure HGS using the dominant hand, while the participant was sitting and the elbow was at 90° of flexion. The protocol for measuring muscle strength was based on the Southampton method. In this method, the person is asked to sit in a comfortable and standard chair, while the dominant arm is fixed at 90° on the handle of the chair. In this work, the participants were asked to position the hand so that the thumb was round one side of the handle, and the four fingers were around the other side. After that, the participants were asked to squeeze the handle with maximal effort for 10 s, then dynamometry was repeated after 30 s, and their average was reported as HGS. The calibration of this dynamometer was performed according to the manufacturers’ manual. This device was calibrated at the factory by loading it at the center with weight and making appropriate adjustments in the gauge. The calibration should be checked once a year. If the device is dropped or there is some particular reason to suspect that the calibration is in error, the device should be recalibrated for which it is recommended to be returned to SAEHAN Corporation. The validity and reliability of this device were confirmed by Reis et al. [21].

#### Outcome measurement

Low back pain and arthralgia were surveyed using participants’ self-reports about pain, as well as the pain area based on the RaNCD cohort study physician opinion and participants’ response to her questions as follows: 1) Have you ever had a low back pain lasting more than a week and resulting in serious disruption in your daily activity? (Yes/No); 2) Do you have a history of back stiffness for more than an hour in the morning? (Yes/No); 3) Do you have a history of arthralgia? (Yes/No); 4) Do you have a history of joint stiffness for more than an hour in the morning? (Yes/ No). These questions were administered by the PERSIAN mega cohort study to evaluate chronic diseases in all Iranian adults ages≥35 years. Then, participants with low back pain and arthralgia were specifically examined by their physician and pain associated with malignancies, infections, and fractures were not considered low back pain. Therefore, the RaNCD cohort study physician diagnosed the type of musculoskeletal pain in the participants based on their medical history and musculoskeletal pain self-report.

#### Statistical analysis

Stata, version 14 (Stata Corp, College Station, TX) was applied for all statistical analysis. We compared baseline characteristics of studied participants by Chi-square and independent samples T-test. Quantitative variables were presented as mean ± standard deviation. Further, qualitative variables were reported using frequency (%). In order to assess the relationship of muscle strength with back pain and arthralgia, logistic regression was performed to produce odds ratios (OR) for binary outcomes in crude and adjusted models (stepwise). We considered the variables of age and physical activity in adjusted Model 1. Furthermore, in adjusted Model 2, in addition to the variables of Model 1, we controlled the variables of drinking and diabetes. Further, to better illustrate this association, we considered linear regression OR across increased muscle strength with adjustment for the mentioned variables in logistic regression. *P*-values were considered significant at the level of < 0.05.

## Results

### Participants and descriptive data

In the current study, 2164 of RaNCD participants met the inclusion criteria and thus included in. Weight mean showed no difference in all participants with and without low back pain and arthralgia (*P* = 0.326 and 0.87, respectively). Mean HGS in participants without arthralgia was significantly higher than those with (*P* < 0.001), while there was no HGS difference in participants with and without low back pain (P = 0.326). 21.39 and 24.58% of studied participants reported low back pain and arthralgia. Among the participants with low back pain, 14.5% had back stiffness, and among those with arthralgia, 12.8% had joint stiffness. The baseline characteristics of the studied participants are presented in Table 1.

**Table 1:** Baseline characteristics of studied participants

Variables	Total (*n* = 2164)	Without low back pain (*n* = 1701)	With low back pain (*n* = 463)	P**	Without arthralgia (*n* = 1632)	With arthralgia (*n* = 532)	P**
Age (year)	46.77 ± 7.89^a^	46.53 ± 7.86	47.63 ± 7.91	0.006	46.21 ± 7.71	48.48 ± 8.17	< 0.001
Weight (kg)	75.44 ± 13.31	75.31 ± 13.23	75.92 ± 13.61	0.326	75.38 ± 13.10	75.63 ± 13.95	0.87
BMI (kg/m^2^)	25.94 ± 4.07	25.96 ± 4.02	25.91 ± 4.25	0.05	25.87 ± 4.04	26.15 ± 4.16	0.432
WC (cm)	96.32 ± 9.73	96.35 ± 9.2	96.22 ± 11.46	0.505	96.06 ± 9.81	97.12 ± 9.43	0.03
Muscle strength (kg)	41.15 ± 9.27	41.23 ± 9.24	40.83 ± 9.42	0.326	41.69 ± 9.17	39.47 ± 9.4	< 0.001
PA (MET hour/day)	43.21 ± 9.65	43.27 ± 9.77	43.01 ± 9.18	0.893	43.17 ± 9.63	43.34 ± 9.71	0.472
Drinking, %	13.7	13.6	14	0.436	13	16	0.05
Diabetes, %	6.3	6.6	5.4	0.218	5.8	7.9	0.05
Back stiffness, %	5.1	2.5	14.5	< 0.001	2.6	12.8	< 0.001
Joint stiffness, %	3	2.1	6	< 0.001	0.4	10.9	< 0.001

*BMI* Body mass index, *WC* Waist circumference, *PA* Physical activity

**P-values were obtained independent samples T test and Chi square

^a^Mean ± SD

### Main results

**Fig. 2:**
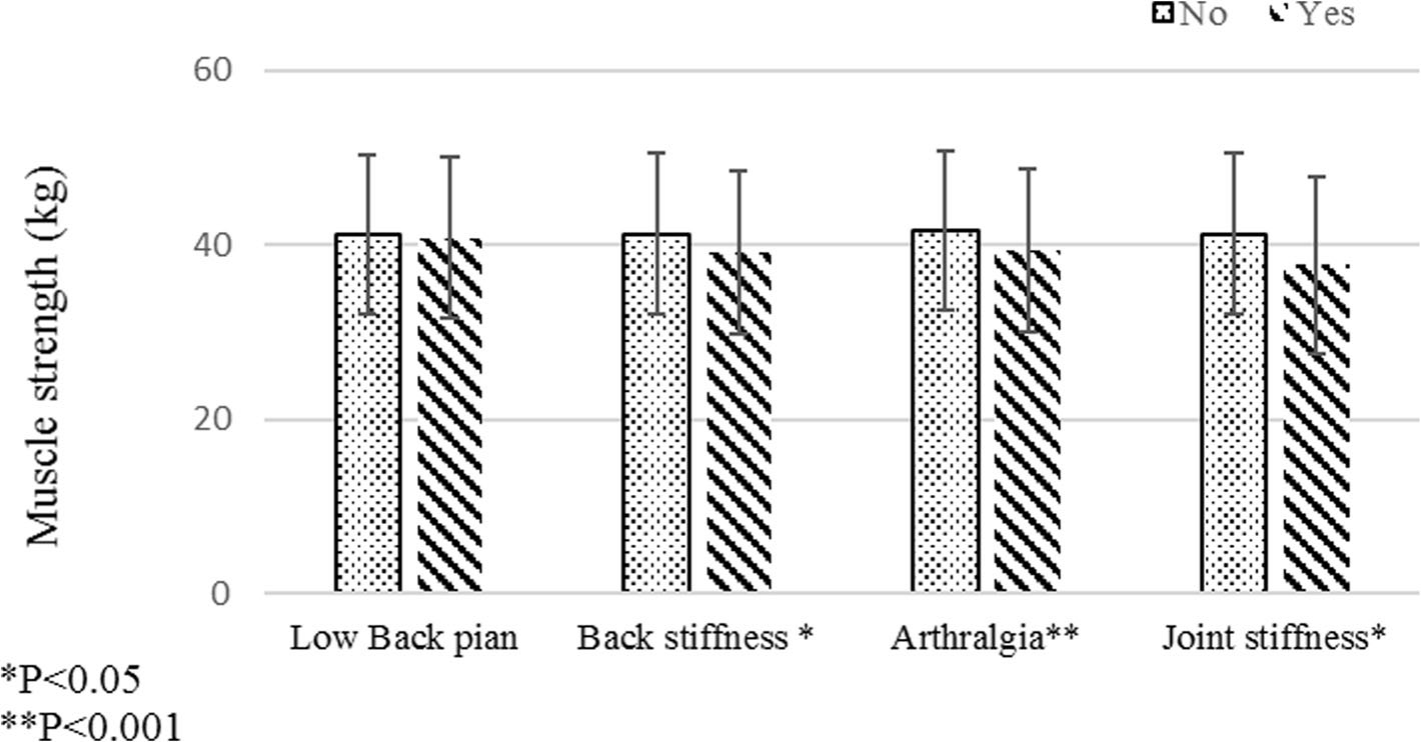
Differences of muscle strength in participants with low back pain, back stiffness, arthralgia, and joint stiffness and without of all them (*P = 0.326; *P* = 0.05; *P* < 0.001; and *P* = 0.005, respectively)

Figure 2 shows that HGS mean in participants with arthralgia and back & joint stiffness is significantly less than that in those without these disorders (P < 0.001, *P* = 0.05, and *P* = 0.005, respectively).

Multiple-adjusted OR and 95% confidence intervals (CI) for arthralgia and back & joint stiffness across muscle strength showed that increasing HGS was associated with a lower risk of their occurrence (Table 2). These associations remained after adjusting for potential confounders, including age, physical activity, drinking, and diabetes. According to the findings, participants with better HGS were 2% less likely to have arthralgia. Better muscle strength was also associated with a 3 and 4% reduced risk of back and joint stiffness (Table 2).

**Table 2:** Relationship between muscle strength and back and arthralgia

	**Low back pain**	**P**	**Arthralgia**	**P**
Crude	0.99 (0.98–1)	0.371	0.97 (0.96–0.98)	< 0.001
Model 1^a^	1 (0.99–1.01)	0.744	0.98 (0.97–0.99)	0.012
Model 2^b^	1 (0.98–1.01)	0.796	0.98 (0.97–0.99)	0.008
Crude	**Back stiffness**	**P**	**Joint stiffness**	**P**
Model 1^a^	0.97 (0.95–0.99)	0.013	0.95 (0.93–0.98)	0.002
Model 2^b^	0.98 (0.96–1)	0.098	0.97 (0.94–1)	0.077
	0.97 (0.95–0.99)	0.048	0.96 (0.93–0.99)	0.049

^a^Model 1 adjusted for age and physical activity

^b^Model 2 adjusted for variables in model 1, drinking, and diabetes

**Fig. 3:**
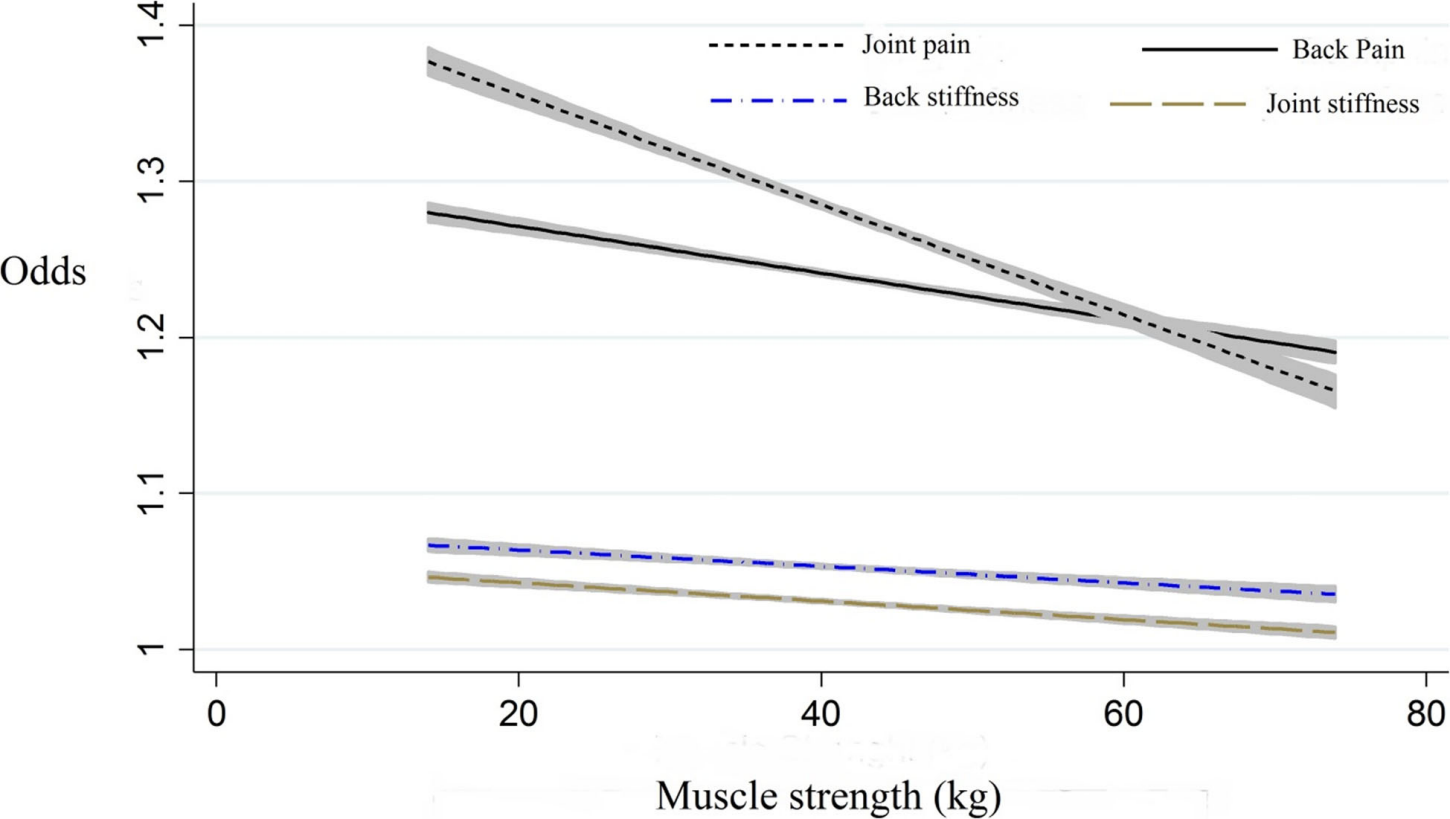
Liner regression between muscle strength and low back pain and arthralgia

Moreover, results from multivariable logistic regression showed that risk of back pain, arthralgia, as well as back and joint stiffness, decreased with increasing HGS after adjusting for potential confounders, as shown in Fig. 3.

## Discussion

Our findings revealed that higher HGS could decrease the risk of arthralgia, as well as back and joint stiffness. Indeed, HGS is a general result of the strength of the body used in epidemiological studies [11, 22]. Although the prevalence of musculoskeletal pain in men is lower than women, men are affected by comorbidities associated with these chronic pains as well, leading them to risky behaviors to suppress pain, such as the use of narcotic drugs [23]. Low muscle strength can predict frailty, sarcopenia, falls, fractures, and overall reduced quality of life [12, 24]. HGS is a valid and reliable method for determining the maximum hand muscle strength that shows overall muscle strength and nutritional status [13]. Since musculoskeletal pain is associated with disability and inactivity, recognizing the link between muscle strength and these chronic pains can be an appropriate strategy to reduce it. Furthermore, daily and occupational activity may be restricted by musculoskeletal pain.

In the present study, no association between low back pain and HGS was observed. However, higher HGS was associated with a lower risk of stiffness. Alperovitch-Najenson et al. [25] observed that women working in an industrial environment with musculoskeletal pain had low HGS. A study on old women without physical activity showed low HGS to be related to a higher risk of low back pain (*P* = 0.004) [13]. Hershkovitz et al. [22], in their research, indicated that higher HGS was associated with better rehabilitation in patients with hip fracture. Ishak et al. observed that women with low back pain had poorer HGS compared to those of healthy (*P* = 0.04), while they did not find any association between HGS and low back pain in men (*P* = 0.834) [26]. Findings of another cross-sectional study showed a relationship between muscle strength and low back pain in female health care staff, although this pain did not limit their work activity [27]. A clinical trial designed by Han et al. [28] showed that increased muscle strength in lumbar after intervention by aquatic exercise significantly reduced low back pain in older women. Results of a meta-analysis by de Sousa on 14 studies showed the strength of hip abductors, hip extensors, and knee extensor muscles in patients with low back pain to be significantly lower than healthy participants; however, there was no difference in knee flexor muscle strength in patients with low back pain and healthy participants [29]. In the present study, we found no difference in weight, level of physical activity, diabetes, and alcohol consumption in participants with and without back pain. It seems that the lack of HGS difference in participants with and without back pain is due to the effect of these factors, which is similar in both groups. Another possible reason for the lack of association between low back pain and HGS appears to be that people do their daily activities regardless of the pain in the lower back, especially in men.

The results of this study showed that HGS of participants with arthralgia and joint stiffness was significantly low compared to healthy participants. Terauchi et al. [30], in a survey of middle-aged women, showed that a greater HGS was associated with lower muscle and joint pains (OR: 0.92; CI 95%: 0.87–0.97). Forechi et al. [24] observed that patients with post-chikungunya chronic arthralgia had significantly lower HGS than adults without this disorder (*P* < 0.001). Findings of a prospective study on young men showed that decreased muscle strength was not associated with an increased risk of fracture musculoskeletal pain. However, after 17 years of follow-up, musculoskeletal pain was less reported in people with better muscle strength [31].

With aging, the balance between antioxidant capacity and the production of radical oxygen species is disturbed [32, 33]. Further, systemic inflammation, like what occurs in arthritis and other chronic musculoskeletal pain, results in producing inflammatory cytokines, such as plasma interleukin 6 and tumor necrosis factor-alpha [33]. Poor muscle strength may be induced by oxidative stress and inflammatory cytokines production [32]. Excess radical oxygen species and inflammatory cytokines contribute to activating muscle proteases, leading to protein breakdown [32]. Overall, poor muscle strength increases age-related problems, including falls, disability, and mortality [34].

Some studies have divided the stages of the low back pain based on self-report pain intensity into chronic, acute, and subacute. However, they have not evaluated the relationship between muscle strength and stages of low back pain [35–37]. In the RaNCD cohort study, pain intensity was not assessed, and we were unable to perform this classification. However, our findings indicated that optimal HGS could decrease the risk of arthralgia, back pain, and joint stiffness in the Kurdish men population.

### Limitations

This present study was the first on a large sample size in Kurdish men to evaluate HGS and the risk of low back pain and arthralgia. However, this study suffered from several limitations. First, the degree and severity of pain in the cohort study were not measured. Second, this was a cross-sectional study; therefore, it is not possible to infer that increased HGS causes arthralgia, back pain, and joint stiffness or contrariwise. Since our study was conducted only on men, its findings cannot be generalized to society as a whole. Also, the method of evaluating the outcome was self-reporting and can interfere with the findings. Therefore, further studies are necessary without these limitations.

## Conclusions

To sum up, our findings highlighted that better HGS was associated with a lower risk of arthralgia, as well as back and joint stiffness among Kurdish men. However, these findings did not support the role of muscle strength in low back pain. Nevertheless, after controlling for potential confounders, we did not observe any association between muscle strength and low back pain.

## Data Availability

Data will be available upon request from the corresponding author.

## Abbreviations

HGS: Handgrip strength
NCDs: non-communicable diseases
RaNCD: Ravansar non- communicable diseases
PERSIAN: Prospective Epidemiological Research Studies in Iran
BMI: Body mass index
OR: odds ratios
CI: confidence intervals
